# Vascular heterogeneity and targeting: the role of YKL-40 in glioblastoma vascularization

**DOI:** 10.18632/oncotarget.5943

**Published:** 2015-10-01

**Authors:** Rong Shao, Sherry L. Taylor, Dennis S. Oh, Lawrence M. Schwartz

**Affiliations:** ^1^ Department of Biology, University of Massachusetts, Amherst, MA, USA; ^2^ Molecular and Cellular Biology Program, Morrill Science Center, University of Massachusetts, Amherst, MA, USA; ^3^ Department of Neurosurgery, Tufts University, Boston, MA, USA; ^4^ Department of Surgery, Baystate Medical Center, Tufts University, Springfield, MA, USA

**Keywords:** glioblastoma, YKL-40, glioblastoma stem-like cells, transdifferentiation, tumor angiogenesis

## Abstract

Malignant glioblastomas (GBM) are highly malignant brain tumors that have extensive and aberrant tumor vasculature, including multiple types of vessels. This review focuses on recent discoveries that the angiogenic factor YKL-40 (CHI3L1) acts on glioblastoma-stem like cells (GSCs) to drive the formation of two major forms of tumor vascularization: angiogenesis and vasculogenic mimicry (VM). GSCs possess multipotent cells able to transdifferentiate into vascular pericytes or smooth muscle cells (PC/SMCs) that either coordinate with endothelial cells (ECs) to facilitate angiogenesis or assemble in the absence of ECs to form blood-perfused channels via VM. GBMs express high levels of YKL-40 that drives the divergent signaling cascades to mediate the formation of these distinct microvascular circulations. Although a variety of anti-tumor agents that target angiogenesis have demonstrated transient benefits for patients, they often fail to restrict tumor growth, which underscores the need for additional therapeutic tools. We propose that targeting YKL-40 may compliment conventional anti-angiogenic therapies to provide a substantial clinical benefit to patients with GBM and several other types of solid tumors.

## INTRODUCTION

Glioblastoma (GBM), a grade IV glioma, is the most lethal primary brain tumor in human with a median survival of around 3 months without treatment and 12-15 months with therapeutic interventions [1-2]. Even with extensive surgical excision and post-operative adjuvant radio/chemotherapy, approximately half of patients relapse, and fewer than 3% of cancer patients survive longer than 5 years [3–4]. Although GBMs rarely spread outside the central nervous system, they typically present as extensive infiltrating tumors with the ability to vigorously invade adjacent normal brain tissue, thereby precluding curative surgical removal. GBM is characterized by strong vascular proliferation that is associated with tumor cell growth, invasion, resistance to chemo/radiotherapy, and decreased disease-free survival [5–6]. Hence understanding the molecular mechanisms that mediate vascular development and pathogenesis is of paramount importance in clinical practice for patients who receive anti-angiogenic drug therapy.

A number of distinct vascular phenotypes in GBM have been identified in which tumor angiogenesis is one of the most prominent forms of vascularization [7–8]. Tumor angiogenesis is a vessel-sprouting process characterized by the migration and proliferation of pre-existing vascular endothelial cells (ECs) followed by the recruitment of pericytes or smooth muscle cells (PC/SMCs) that support vessel stability and enable blood perfusion [9]. Some of the pre-existing vessels are also able to split and give rise to secondary daughter vessels, a vascular event known as intussusception [8]. Vessel co-option also occurs in some cases, whereby tumor cells drive or hijack pre-existing vessels in order to develop new vascular networks [10–11]. Over the past a few years, another major form of tumor vascularization has been discovered in GBM in which tumor cells assemble to vascular channels, independent of endothelial cells [12–13]. Such tumor cell-mediated vascular formation lacking ECs is referred to as vasculogenic mimicry (VM) [14].

Over the past decade, the secreted glycoprotein YKL-40, also named chitinase 3-like 1 (Chi3l1) [15–18], has emerged as a potential mediator of GBM progression. YKL-40 is a highly conserved 40-kDa chitin- or heparin-binding glycoprotein, which places it into the family of chitinase-like proteins. However, YKL-40 lacks chitinase/hydrolase activity because of a mutation in the chitinase-3-like catalytic domain that converts an essential glutamic acid into leucine residue. Gene expression profiling has demonstrated that YKL-40 is ranked as one of the most dramatically induced genes in GBM [19–20]. A wealth of clinical evidence has also revealed that elevated serum levels of YKL-40 in GBM are positively correlated with cancer invasiveness, radioresistance, recurrence, and reduced patient survival times [19–25]. In concert with these findings, radiotherapy-resistant GBMs express elevated levels of YKL-40, which may at least partially contribute to the tumor malignancy [21, 26]. The roles and molecular mechanisms that mediate YKL-40-dependent vascularization of GBM have been the subject of several recent studies. We have found that YKL-40 acts as an angiogenic factor to promote tumor angiogenesis in both GBM and breast cancer [27–28]. This review focuses primarily on the recently identified roles of YKL-40 in facilitating both angiogenesis and VM in GBM, with a focus on current therapeutic limitations evident in the treatment of patients with conventional anti-angiogenic drugs.

## 2. TUMOR ANGIOGENESIS

### 2.1. Endothelial cells

Tumor angiogenesis is a pathologic process that is primarily mediated by the growth and sprouting of vascular ECs [29–30]. These ECs typically develop from vascular lineage differentiation of bone marrow-derived CD34^+^-hematopoietic stem cells. Other cell types can also participate in tumor angiogenesis, including endothelial progenitor cells (EPCs) and cancer stem cell-differentiated vascular cells [31–33]. While the majority of glioblastoma stem-like cells (GSCs) commit to neural lineage differentiation including glial cells or astrocytes, neurons, and oligodendrocytes, a small percentage of GSCs display the ability to transdifferentiate into ECs [31, 34–36]. However, the molecular mechanisms underlying the vascular transdifferentiation of GSCs are still poorly understood and its potential clinical importance for tumor vascularization and malignance in patients with GBM has yet to be fully determined.

**Figure 1:**
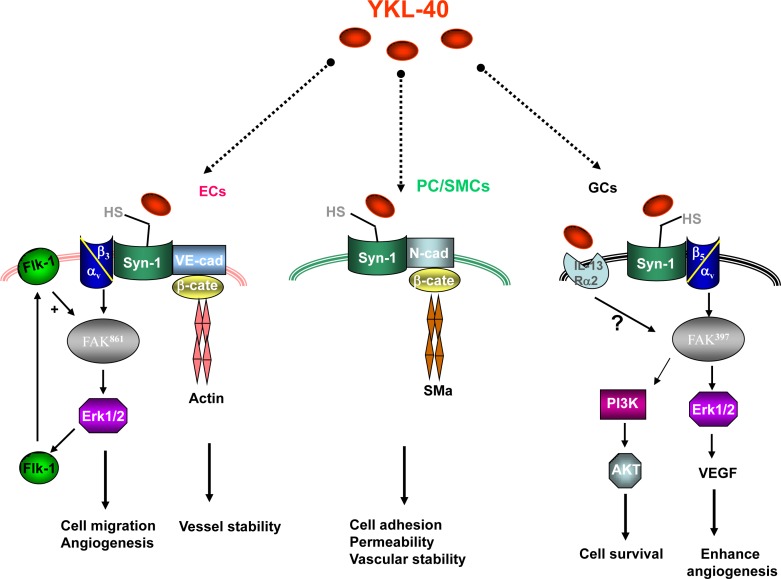
YKL-40 induces multiple signaling pathways in endothelial cells (ECs), pericytes/smooth muscle cells (PC/SMCs), and glioblastoma cells (GCs) YKL-40 activates interaction of syndecan-1 (Syn-1) and integrin αvβ3, which leads to intracellular signaling via FAK^861^ and ERK 1/2 [28], and several tumor-promoting processes that include the expression of VEGFR 2 (Flk-1), enhanced cell motility, and EC-mediated angiogenesis [40]. YKL-40-induced Flk-1 activation enhances angiogenic responses. YKL-40 may prompt coupling of Syn-1 with vascular cadherin (VE-cad), which then recruits β-catenin (β-cate) and cytoskeleton protein actin, thereby facilitating vessel stability [60]. YKL-40 can also induce the association of Syn-1 with N-cadherin (N-cad) and intracellular β-cate and downstream smooth muscle alpha actin (SMa) in PC/SMCs, which leads to increased cell adhesion, enhanced permeability, and vascular stability [60, 63]. Identical to angiogenic signaling in ECs, YKL-40 stimulates coordination of Syn-1 and integrin αvβ5, downstream effectors FAK^397^ and ERK 1/2, which induces the expression of VEGF in glioblastoma cells (GCs) [27]. In addition, YKL-40 augments signaling cascades PI3K-AKT, which in turn protects GCs from γ-irradiation-induced apoptosis. Intracellular signaling pathways for YKL-40-binding receptor IL-13Rα2 in GCs need to be established. HS: heparan sulfate chains that bind to YKL-40.

While there is a substantial body of research characterizing the role of angiogenic factors like vascular endothelial cell growth factor (VEGF) in facilitating vascularization of GBM (see multiple excellent review articles [3, 37–38]), it has been demonstrated recently that YKL-40 also plays pivotal roles in GBM [27]. YKL-40 is a potent angiogenic factor that is able to induce endothelial cell angiogenesis. Recombinant YKL-40 can promote tube formation and migration of cultured ECs with the same angiogenic potential as VEGF, one of the most potent angiogenic factors yet identified [28, 39]. In addition, YKL-40 induces both VEGF expression in a GBM-derived cell line U87 and VEGF receptor 2 (VEGFR 2) expression in ECs (Figure 1) [27, 40]. Consequently, all of these angiogenic molecules may collaborate synergistically to trigger tumor angiogenesis. Interestingly, inhibition of VEGF led to the induction of YKL-40 in U87 cells [25, 27], suggesting potential compensatory effects among multiple angiogenic factors in order to sustain vessel formation. RNAi-mediated gene knockdown of YKL-40 expression in U87 cells significantly inhibited tumor angiogenesis in xenografted animal models, as EC-lined vessel density of YKL-40 shRNA tumors was decreased to 44% of control tumor vessels [28]. In line with these findings, treating U87 tumor-bearing mice with a neutralizing anti-YKL-40 antibody (mAY) resulted in abrogation of tumor angiogenesis, reduced distant metastasis, and increased mouse survival [27, 40], all of which underscore an angiogenic signature of YKL-40 in tumor progression. In a small set of patients with GBM, tumor expression of YKL-40 was correlated with increased EC-associated vessel density and VEGF expression, and decreased patient survival [27], which agrees well with the data in pre-clinical studies. All of these data support the hypothesis that YKL-40 acts as a potent angiogenic factor to stimulate angiogenesis in GBM. It is intriguing to speculate that combined anti-angiogenic therapies targeting both YKL-40 and VEGF might dramatically reduce tumor angiogenesis and block tumor progression.

One candidate “receptor” that may transduce YKL-40-mediated angiogenic activity in ECs is the membrane-bound protein syndecan-1, an abundant cellular surface heparan sulfate (Figure 1) [28, 39]. Given its inherent heparin-binding property, YKL-40 was found to bind to heparan sulfate chains of syndecan-1 on cell surface and facilitate the coupling of syndecan-1 with an adjacent membrane-associated protein integrin αvβ3, thus activating angiogenic responses through FAK^861^ to MAP kinase ERK 1 and 2 in ECs [28]. VEGFR 2 induced by YKL-40 could sensitize ECs to VEGF, resulting in enhanced angiogenesis. Likewise, YKL-40 could induce VEGF expression in U87 cells by means of the similar signaling pathway (Figure 1) [27]. For example, YKL-40 stimulates the association of syndecan-1 with integrin αvβ5, which leads to downstream activation of FAK^397^ and ERK 1 and 2, thereby augmenting VEGF gene expression, which in turn cooperates with YKL-40 to activate ECs and elicit angiogenesis (Figure 1). In addition, YKL-40 has the ability to activate PI_3_K-AKT pathways in U87 cells, which then inhibits cell death induced by γ-irradiation [27, 40]. These mechanistic insights support the hypothesis that YKL-40 functions as both a potent angiogenic factor and a growth factor that serves to promote a number of downstream signaling cascades in both ECs and tumor cells. Jack Elias' group recently identified a YKL-40-binding receptor, IL-13Rα2, which is expressed by macrophages responsible for bacterial killing [41]. Both YKL-40 and IL-13Rα2 were also found to mediate melanoma lung metastasis [42]. In GBM, the expression of IL-13Rα2 was significantly elevated, but it has not been determined yet if IL-13Rα2 functions to mediate YKL-40-induced angiogenesis (Figure 1) [43–44]. It is noted that IL-13Rα2 also serves as a decoy receptor for IL-13 that regulates apoptosis. Hsi et al. found that knockdown of the IL-13Rα2 gene in GBM cells promoted IL-13-dependent cell death and restricted tumor growth [45]. Therefore, it would be valuable to determine if IL-13Rα2 acts as a core factor to control the dual axes of YKL-40/IL-13Rα2 and IL-13/IL-13Rα2 in tumor malignancy.

### 2.2. Pericytes/smooth muscle cells

Once endothelial cells have transformed into vascular tubes, mesenchyme-derived mural cells, referred to as pericytes/smooth muscle cells (PC/SMCs), are recruited to the out layer of the neovessels [46]. The recruitment of PC/SMCs in turn enhances EC proliferation, survival, migration, differentiation, and vascular branching [47–48]. This reciprocal activation is accomplished by the interaction and activation between angiogenic molecules secreted from ECs and their corresponding binding receptors expressed by PC/SMCs, such as angiopoietin-1/Tie-2 [49–50] and platelet-derived growth factor-B (PDGF-B)/PDGF receptor-β (PDGFR-β) [51–53], all of which enable longitudinal spreading of PC/SMCs along EC-based growing vessels. In GBM, it is also notable that PC/SMCs play a pivotal role in the vessel development, as multiple agents targeting PDGF-B/PDGFR-β signaling have been utilized in pre-clinical and clinical trials to inhibition of tumor angiogenesis. However, targeting PDGF could also impair vessel stability and integrity, leading to blood diffusion into the tissue. For example, multiple clinical trials with imatinib that blocks kinase activity of PDGFR and c-kit, have given rise to severe complications that are commonly associated with tumor hemorrhage in young patients with neuroblastoma, GBM and gastrointestinal stromal tumors [54–57] (see following Sections 4.1 & 4.2).

Recently, VEGF was shown to function as a “permeability factor” that impairs PC/SMC-associated vascular integrity. Ablation of myeloid cell-derived VEGF in mice led to increased vessel coverage of PC/SMCs and acceleration of tumorigenesis [58–59]. It would be valuable to determine if this VEGF-inhibited vessel stability depends on PC/SMC expression of VEGFR 1 or 2. In contrast to VEGF, YKL-40 maintains vascular stability and integrity [60]. YKL-40 is expressed by PC/SMCs and serves as a mesenchymal cell marker [61–62]. YKL-40 can promote the association of syndecan-1 with neural cadherin in the cell membrane, which in turn recruits both β-catenin and cytoskeleton protein smooth muscle actin (SMa), further facilitating inter-PC/SMC adhesion (Figure 1). In addition, YKL-40 induces the same coordination of vascular cadherin and β-catenin on ECs, which also contributes to vascular permeability and stability (Figure 1) [60]. Blockade of YKL-40 by either gene knockdown or the use of mAY decreases vessel coverage by PC/SMCs and results in increased vascular permeability and leakage, thus leading to vessel collapse and dysfunction in brain tumor xenografts [60]. Furthermore, combination therapy with mAY and ionizing irradiation synergistically inhibited PC/SMC-mediated tumor angiogenesis [63]. Collectively, these data suggest that YKL-40 acts comparably in PC/SMCs and ECs in ways that govern vascular stability and integrity in GBM. Supporting this hypothesis, a pilot study of ten patients with GBM has revealed a strong correlation between YKL-40 expression on PC/SMCs and tumor vascular stability, permeability and decreased patient survival [60].

## 3. VASCULOGENIC MIMICRY

**Figure 2:**
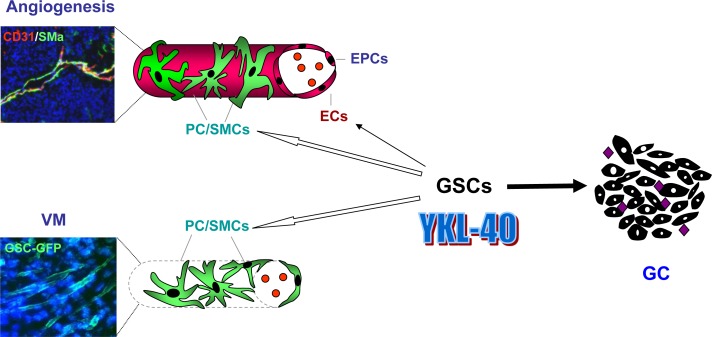
A model for YKL-40-mediated tumor vascularization that is associated with vascular transdifferentiation of GSCs in GBM YKL-40 expressed and secreted by glioblastoma cells (GCs) is associated with tumor vascularization and malignancy of GBM, in which a large population of GCs is derived from a hierarchy of glioblastoma stem-like cells (GSC). GSCs can transdifferentiate into vascular pericytes/smooth muscle cells (PC/SMCs) that support endothelial cell (EC)-based vessel integrity in angiogenesis and as well as assemble into vascular channels in the absence of ECs, a process known as vasculogenic mimicry (VM). In addition, a small population of GSCs also displays the ability to transdifferentiate into ECs that can participate in angiogenesis. In some cases, endothelial progenitor cells (EPCs) cooperate with ECs to develop tumor vessels. Co-immunofluorescence staining shows ECs and PC/SMCs that are specifically recognized by anti-CD31 (red) and smooth muscle actin alpha (SMa) (green) antibodies, respectively. Autofluorescence of GSCs expressing green fluorescent protein (GFP) displays vascular channels in VM. YKL-40 (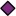) and blood cells (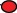).

Separate from the EC-associated angiogenesis, a number of independent studies suggest that VM also contributes to GBM vascularization [64]. This agrees well with the observation that VM is a common microvascular circulation in other cancers such as melanoma, colorectal cancer, and breast cancer [65–67]. VM-associated vasculature can represent up to 50% of total vessel content in some cases of GBM [68]. These vascular-like channels can be formed independently of ECs via transdifferentiation of GSCs into PC/SMCs [69–70]. Although the individual contributions of subpopulation of CD133^+^ and CD133^−^ GSCs into VM are still controversial [71–72], a significant population (~20%) of general GSCs derived from patients with GBM were able to transdifferentiate into PC/SMCs that could participate in both VM and angiogenesis (Figure 2) [12, 68]. GBM-derived PC/SMCs developed VM that lack ECs and exhibited a leaky vascular phenotype [60]. In contrast, in the presence of ECs these GSC-differentiated PC/SMCs can interact intimately with ECs to facilitate angiogenesis and produce vessels that are more stable and had the ability to perfuse tumors more efficiently (Figure 2) [60]. In additional to YKL-40, VEGFR2 was also found to mediate the transdifferentiation of GSCs into PC/SMC and control VM. For instance, VEGFR2 gene knockdown or treatment with a VEGFR2 kinase inhibitor (SU1498) impeded GSC transdifferentiation and subsequent VM in xenograft models and in cultured cells [73–74]. It is noteworthy that VEGFR2-mediated PC/SMC transdifferentiation is independent of VEGF, in contrast with the transdifferentiation of GSCs into ECs which is dependent on VEGF [8, 75–76]. This finding may account, at least partially, for the observation that some recurrent cases of GBM are unresponsive to VEGF-directed drugs such as bevacizumab (see discussion below). Consequently, substantial data support the notion that VEGFR2, like YKL-40, may be an appropriate target for the treatment of GBM.

## 4. ANTI-VASCULAR THERAPIES IN GBM

### 4.1. Anti-angiogenic therapy

Many of the drugs that are used in clinical trials for GBM are designed to inhibit EC and/or PC/SMC receptor tyrosine kinases with the goal of disrupting tumor vasculature (Table 1). For example, FDA approved drugs for the treatment of recurrent GBM, including small molecule inhibitors of the VEGFR kinase and anti-VEGF antibodies like bevacizumab, target the VEGF pathways (Table 1). As reviewed in a number of previous publications, most of clinical trials exploiting these agents have provided encouraging results such as increased progression-free survival in patients (Table 1) [3, 77–80]. Accordingly, multiple research labs have also employed some of these drugs, or related compounds, in orthotopic xenografted animal models in order to validate these clinical findings (Table 2) [81–83]. Despite the promising evidence documented in the literature, the overall benefits of these treatments for patients with GBM are moderate and transient, as high mortality of the disease remains static.

### 4.2. Vessel normalization

Pre-clinical and clinical studies have established a new therapeutic paradigm that is complementary to the conventional vessel-blocking regimens, in which normalization of tumor vascular abnormalities impedes tumor development [84–85]. Instead of a complete blockade of tumor vessel formation, some of anti-angiogenic therapies, including VEGFR kinase inhibitors cediranib and SU5416, an anti-VEGFR antibody DC101, and a PDGFR kinase inhibitor SU6668, ameliorate vascular normalization and alleviate peritumoral edema in GBMs and other cancers (Table 1 & 2) [86–90]. PC/SMCs act as the primary cells that support vessel function and blood perfusion. Consequently, depletion of PC/SMCs in animal tumor models diminished aberrant blood vessel formation, increased tumor hypoxia, and ultimately restrained tumor growth [9]. In agreement with these animal studies, over-expression of PDGF in patient-derived tumors was found to be associated with enhanced cancer malignancy [91], while blockade of PDGF signaling resulted in improvement of drug delivery and chemotherapy [47, 92]. It should be noted that co-delivery of two different drugs targeting ECs (*e.g.* VEGFR inhibitor) and PC/SMCs (*e.g.* PDGFRβ inhibitor) was more effective in inhibiting tumor development than individual anti-angiogenic drugs, presumably because they enhanced the destabilization of vessels by inhibiting PC/SMC function, thus rendering ECs more susceptible to EC blockers [93–94]. In spite of these encouraging findings, conflicting evidence has also been reported from several animal studies and clinical trials. For instance, deletion of PC/SMCs promotes tumor progression, possibly due to lack of a barrier that prevents tumor cells from dissemination into the circulatory system [95]. Consistent with these findings, decreased PC/SMC coverage around vessels in patients with colorectal cancer is correlated with enhanced cancer metastasis [96]. Although the molecular mechanisms underlying these unfavorable outcomes remain to be elucidated, they do suggest that multiple regulatory pathways are likely involved in the formation and maintenance of tumor vascular network that depends on spatial-temporal interaction between ECs and PC/SMCs. In addition, treating GBM patients with imatinib in order to target PDGFR-mediated vessel stability could lead to intra-tumoral bleeding, a severe side effect observed frequently in the anti-vascular therapy [54, 57, 97]. Therefore, the multiple factors that control these cell activities and influence vessel coverage, permeability and stability, oxygen delivery, and blood perfusion should be circumspectly considered in evaluating drug delivery and therapy.

**Table 1:** Anti-vascular agents used in clinical trials of GBM

Name	Molecular targets	Action mechanisms
Aflibercept (VEGF trap)	VEGF-A; VEGF-B, PIGF	Decoy receptor
AMG102	HGF	Anti-HGF antibody
Bevacizumab	VEGF-A	Anti-VEGF antibody
Brivanib	FGFR, VEGFR2	Receptor tyrosine kinase inhibitor
Cediranib (AZD2171)	VEGFR1-3, PDGFRβ, c-kit	Receptor tyrosine kinase inhibitor
Cilengitide	Integrin αvβ3/αvβ5	Short peptides binding integrins
CT-322	VEGFR1-3	Adnectin
Dasatinib	PDGFRβ, Src, BCR-Abl, c-kit, ephrin A2	Receptor tyrosine kinase inhibitor
Erlotinib	EGFR	Receptor tyrosine kinase inhibitor
Gefitinib	EGFR	Receptor tyrosine kinase inhibitor
Imatinib	PDGFRβ, BCR-Abl, c-kit,	Receptor tyrosine kinase inhibitor
Pazopanib	VEGFR1-3, c-kit, PDGFRα/β	Receptor tyrosine kinase inhibitor
Sorafenib	VEGFR2, 3, c-kit, PDGFRβ, Raf	Receptor tyrosine kinase inhibitor
Sunitinib	VEGFR2, 3, c-kit, PDGFRβ, FLT3	Receptor tyrosine kinase inhibitor
Tandutinib	PDGFRβ, FLT3, c-kit	Receptor tyrosine kinase inhibitor
Vandetanib (ZD6474)	VEGFR2, EGFR	Receptor tyrosine kinase inhibitor
Vatalanib	VEGFR1-3, c-kit, PDGFRα/β	Receptor tyrosine kinase inhibitor
XL-184	VEGFR2, c-kit, FLT3, TIE2, c-Met	Receptor tyrosine kinase inhibitor

### 4.3. An unexpected therapeutic outcome −− angiogenic rebound

Multiple independent clinical trials evaluating bevacizumab have demonstrated patient benefits for a number of different cancers, which led to FDA approval of bevacizumab as a first-line treatment for brain tumors [82, 98–100], breast cancers [101], colorectal cancers [102], and non-small-cell lung cancers [103–104]. However, several recent clinical investigations with large patient cohorts suggest that the use of this anti-angiogenic therapy for advanced tumors are controversial and the ultimate benefits are still inconclusive [81, 105–113]. For example, a long-term therapeutic intervention with bevacizumab in GBMs only produced a transitory benefit, with no significantly prolonged overall survival. Once the therapy was terminated, the tumors underwent vascular recovery and regrew rapidly. In concert with these clinical observations, GBM xenografts in mice treated with bevacizumab or DC101 displayed reduced tumor blood supply, but unexpectedly, increased tumor cell invasion [114–116]. In addition, treatment with sunitinib, DC101 and other VEGFR inhibitors (AG-013736 and AG-028262) in other animal tumor studies led to revascularization, increased tumor cell invasiveness, and distant metastasis [116–118]. This rapidly acquired adaptation to anti-angiogenic therapies is recognized to be associated with the angiogenic switch whereby treated tumors undergo robust revascularization and malignant transformation [117, 119]. In addition to the contribution by EC-derived new vessel formation in angiogenic resistance, alternative vascular networks like VM should not be neglected, since tumor-derived vascular channels likely offer a new blood-perfused microcirculatory system. Indeed, PC/SMC-derived vascular channels during VM are resistant to bevacizumab in xenografts [8, 75]. Therefore, it might be quite interesting to determine if tumor cells or tumor-derived PC/SMCs act as the predominant vascular cells to orchestrate neovasculature including vascular channel formation via VM in patients that are resistant to angiogenic drugs. Although the molecular mechanisms underlying this drug resistance remain to be clarified, multifaceted therapies targeting both ECs and PC/SMCs might offer a significant clinical benefit.

**Table 2:** Anti-vascular agents used in glioblastoma xenografts

Name	Action mechanisms	References
Bevacizumab	Anti-VEGF antibody	[83],[115]
Cediranib	VEGFR tyrosine kinase inhibitor	[87]
DC101	Anti-VEGFR2 antibody	[88], [90], [116]
SU10944	VEGFR2 tyrosine kinase inhibitor	[116]
SU5416	VEGFR2 tyrosine kinase inhibitor	[89]
SU6668	PDGFR tyrosine kinase inhibitor	[89]
Sunitinib	VEGFR2 tyrosine kinase inhibitor	[116]

## 5. CHALLENGES AND FINAL REMARKS

Robust vascular proliferation is one of the hallmarks of GBM, in which tumor angiogenesis is commonly recognized as the primary component of tumor vessels. Interestingly, growing evidence suggests that tumor cell-associated vascular channels lacking ECs represent a significant portion of the tumor vasculature in some cases of GBM. Indeed, a simplistic vasculature model merely focusing on EC-mediated angiogenesis is insufficient to describe the range of sophisticated neovascular networks in which tumor cells and bone marrow-derived cells (*e.g.* EPCs, myelomonocytes) also participate in neovascularization [120–122]. In addition to GSC-derived tumor cells, it would be worthy to determine what other cell types also contribute to VM. A growing literature suggests that tumor cell-derived vascular channels represent at least one of the alternative microvascular systems that are independent of EC-associated angiogenesis and that they represent an alternative vascular supply when traditional anti-angiogenic drugs fail in therapy [13, 123–124]. A number of individual research groups have demonstrated that GSCs can transdifferentiate into PC/SMCs and ECs, both of which mediate tumor vascularization [31, 68, 125]. However, many of the details of the underlying cellular and molecular pathways that mediate this transdifferentiation *in vivo* remain to be defined. In particular, we still lack sufficient knowledge of if and how GSC-derived PC/SMCs cooperate with host mesenchyme-derived PC/SMCs to contribute to angiogenesis and VM. Are the former cells more active than the latter during tumor vascularization? If so, do VEGFR, YKL-40, or other factors render these cells more aggressive? Why do GSCs preferentially transdifferentiate into PC/SMCs rather than ECs? What are the core factors that commit GSCs to these individual differentiations into varied types of vascular cells? Understanding these key regulatory processes may help facilitate the identification of new therapeutic targets for cancer treatment, especially for patients that are refractory to anti-EC-directed angiogenic drugs. It is also worth noting that continuously monitoring the dynamic changes in the circulating levels of YKL-40 in patients that receive some of anti-angiogenic drugs might help predict unfavorable outcomes of the disease, and trigger the utilization of alternative therapeutic strategies.

A number of well control studies have reported that bevacizumab fails to yield therapeutic efficacy in blocking tumor vascularization or increasing patient survival. Consequently, identifying the key factors that promote VEGF-independent tumor vascularization is of paramount importance for treatment of recurrent patients that resist bevacizumab. Elevated serum levels of YKL-40 in GBM patients are positively correlated with tumor invasiveness, resistance to chemo/radiotherapy, and short survival, suggesting that YKL-40 serves as a prognostic biomarker for poorer clinical outcomes. YKL-40 stimulates tumor vascularization via EC and PC/SMC-coordinated angiogenesis and PC/SMC-driven VM, thus pointing to YKL-40 as a new target for cancer therapy. A number of independent methods to target YKL-40 activity (*e.g.* YKL-40 shRNA and mAY) have provided constant therapeutic promise in both *in vitro* and *in vivo* studies [39]. Specifically, the anti-vascular efficacy of mAY in animal xenografts suggests a new and exciting therapeutic avenue whereby a humanized anti-YKL-40 antibody could offer a substantial benefit for the treatment of several cancers, resulting in an enhanced quality of life for patients. Anti-YKL-40 therapy may also sensitize tumors to anti-VEGF/VEGFR treatment in relapsing patients that demonstrate resistance to anti-VEGF/VEGFR drugs. In addition, targeting YKL-40 receptor IL-13Rα2 is also anticipated to serve as a potent alternative strategy for either blocking YKL-40-induced tumor vascularization or eliciting a synergetic effect in conjunction with mAY. While a significant population of patients with recurring GBM are resistant to anti-angiogenic drugs, an alternative strategy with combined therapies targeting YKL-40 and other angiogenic factors might represent a significant advance for treatment.

## Abbreviations

GBM: glioblastoma
YKL-40: human cartilage glycoprotein-39 or chitinase-3-like-1
mAY: a neutralizing anti-YKL-40 antibody
ECs: endothelial cells
PC/SMCs: pericytes or smooth muscle cells
GSCs: glioblastoma-stem like cells
VM: vasculogenic mimicry
EPCs: endothelial progenitor cells
VEGF: vascular endothelial cell growth factor
VEGFR: vascular endothelial cell growth factor receptor
PDGF-B: platelet-derived growth factor-B
PDGFR-β: PDGF receptor-β.
